# Supplementary figures and images for: Using B cell receptor lineage structures to predict affinity
Source: PLoS Comput Biol. 2020 Nov 11;16(11):e1008391. doi: 10.1371/journal.pcbi.1008391 (PMC7682889; doi:10.1371/journal.pcbi.1008391)

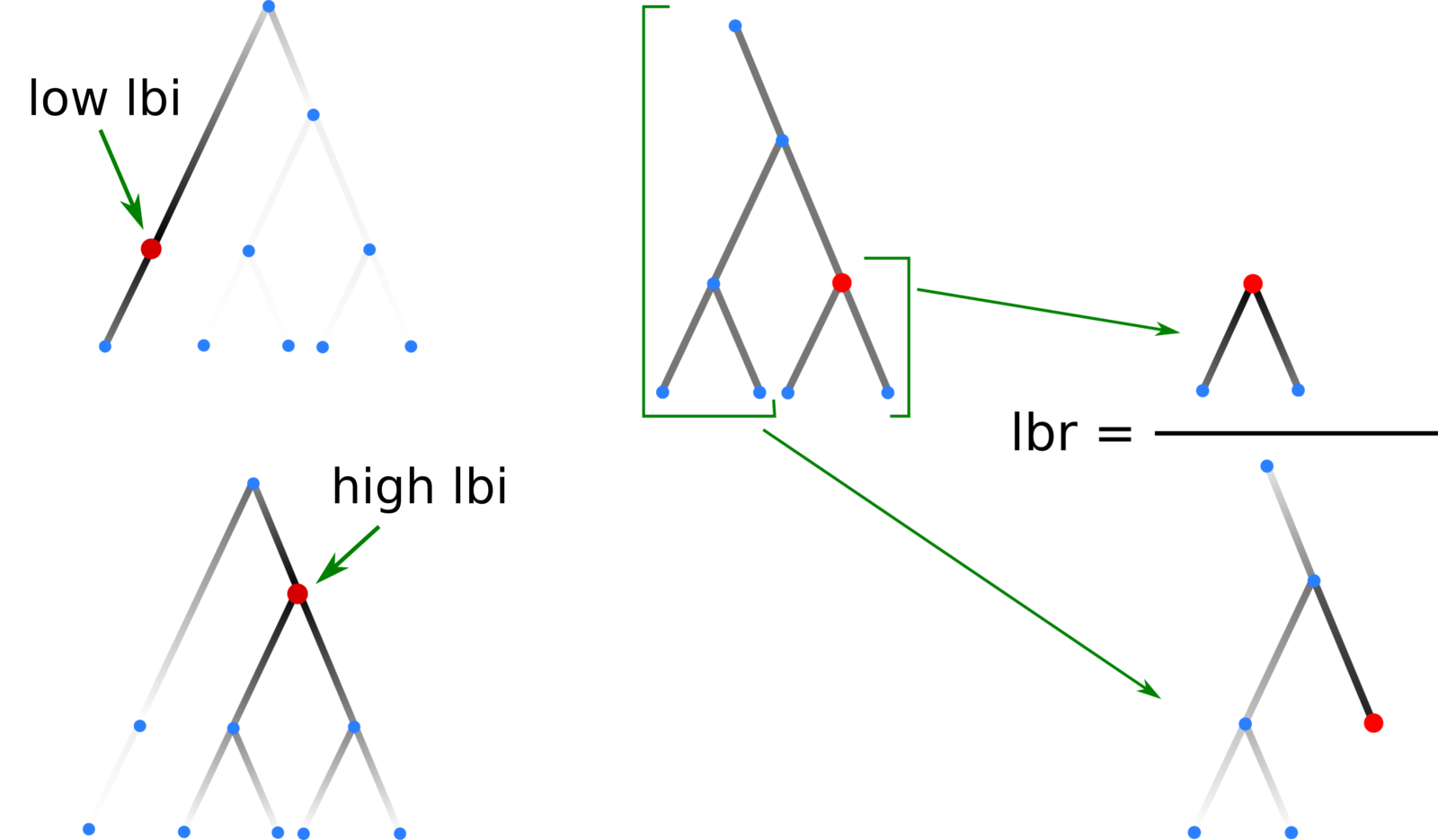

Supplement: S1 Fig — The darkness of each branch represents the exponentially decaying weight factor, which decreases with distance from the node (in red) for which we’re calculating the metric. On the left, we show a node with low nuc-lbi (top) and high nuc-lbi (bottom). At right, in calculating nuc-lbr for the red node, we split the tree into two pieces: offspring of the node in the numerator; and parents, siblings, cousins, and their offspring in the denominator. (TIFF) [file pcbi.1008391.s001.tiff]

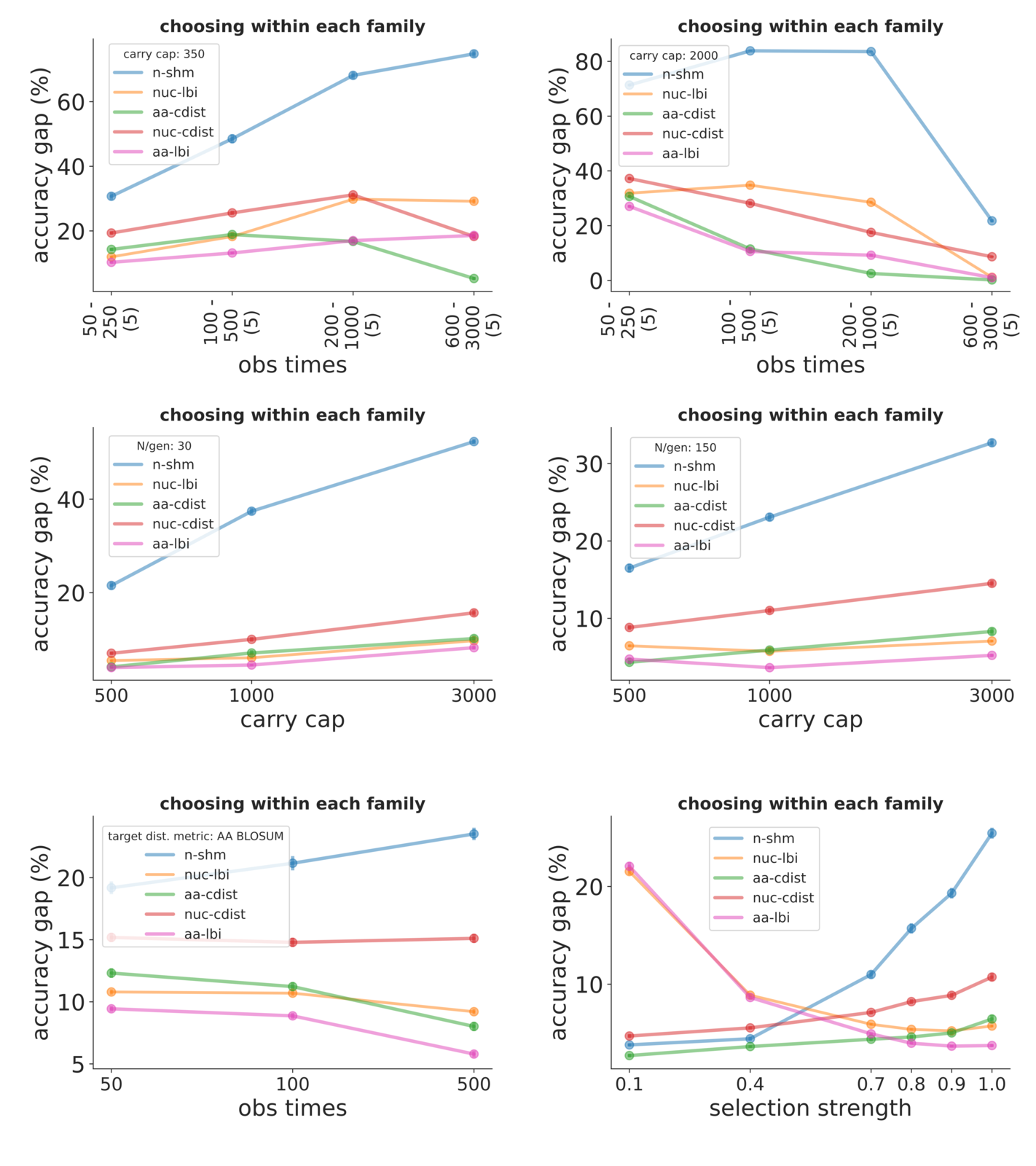

Supplement: S2 Fig — Performance is shown vs observation times (units of N generations), where sampling occurred at five different time points spanning the indicated values for carrying capacity of both 350 (top left) and 2000 (top right). This is in contrast to Fig 1, where sequences were sampled only at the same, final time point. Performance is also shown vs carrying capacity with 30 (middle left) and 150 (middle right) sampled sequences per family; vs observation time for a non-default affinity calculation utilizing BLOSUM matrices (bottom left); and vs a parameter describing the strength of selection (bottom right). The corresponding among-family plots, as well as plots for many other parameter combinations, are in https://zenodo.org/record/3929565. (TIFF) [file pcbi.1008391.s002.tiff]

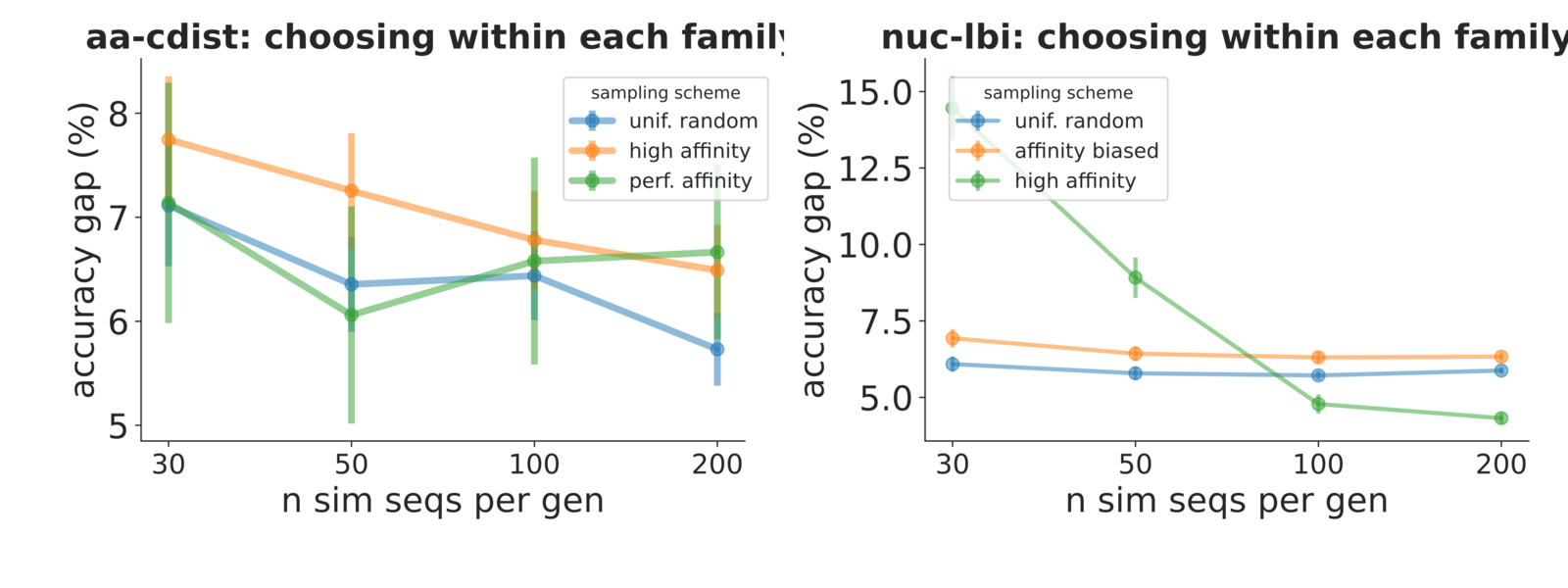

Supplement: S3 Fig — Schemes shown are “uniform random” (the default, which is shown in all other plots), “affinity biased” (the probability of sampling each cell is proportional to its affinity), and “perfect affinity” (sample the N cells with highest affinity). Corresponding among-families plots, and plots for all other metrics, are at https://zenodo.org/record/3929565. (TIFF) [file pcbi.1008391.s003.tiff]

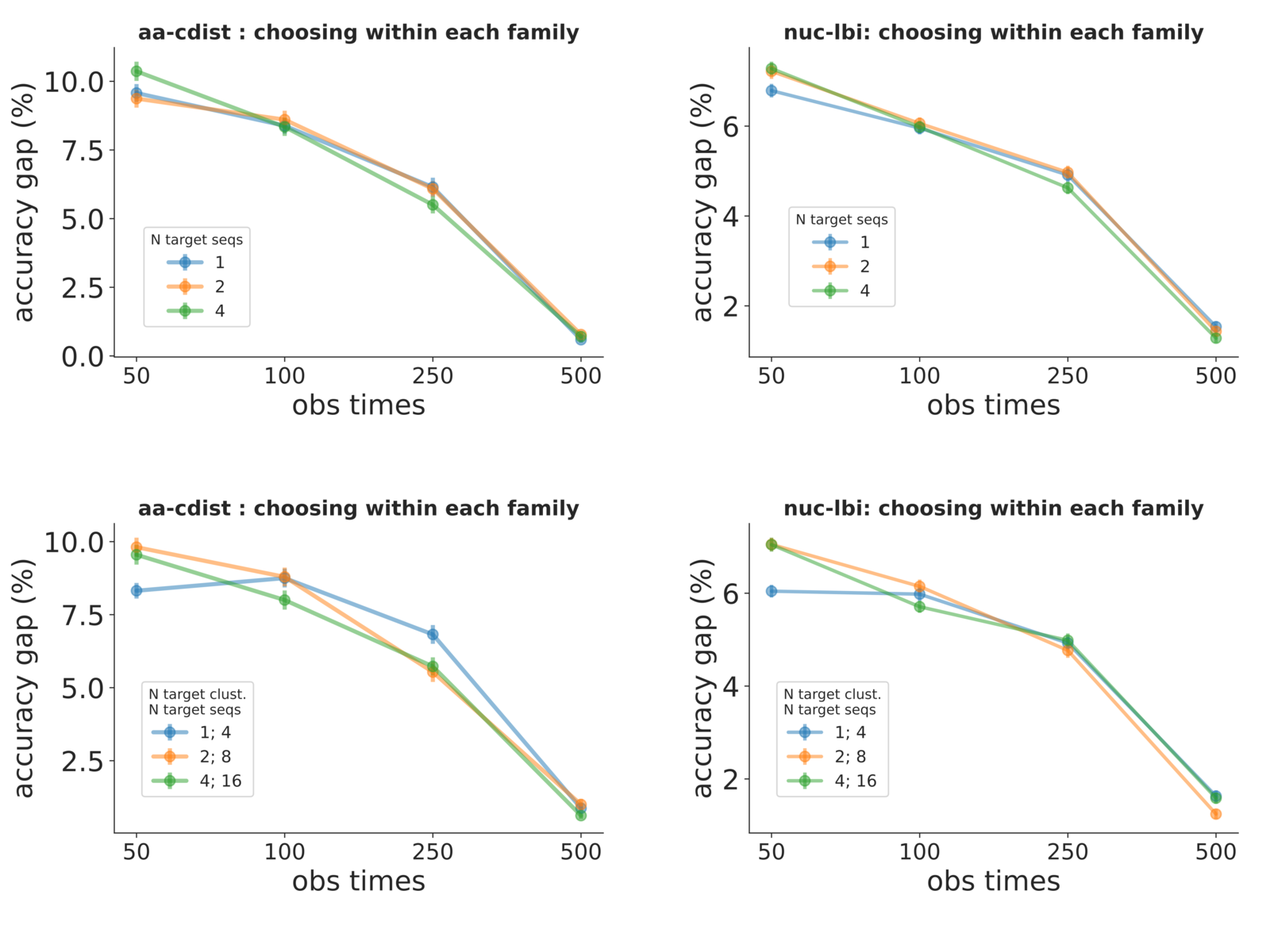

Supplement: S4 Fig — The target sequence represents a hypothetical optimal antibody toward which selection is directing the cells (see text). Shown for 1, 2, and 4 independently-chosen target sequences (top); as well as for 4, 8, and 16 target sequences divided among the indicated number of “clusters” of target sequences (bottom). Corresponding among-families plots, and plots for all other metrics, are at https://zenodo.org/record/3929565. (TIFF) [file pcbi.1008391.s004.tiff]

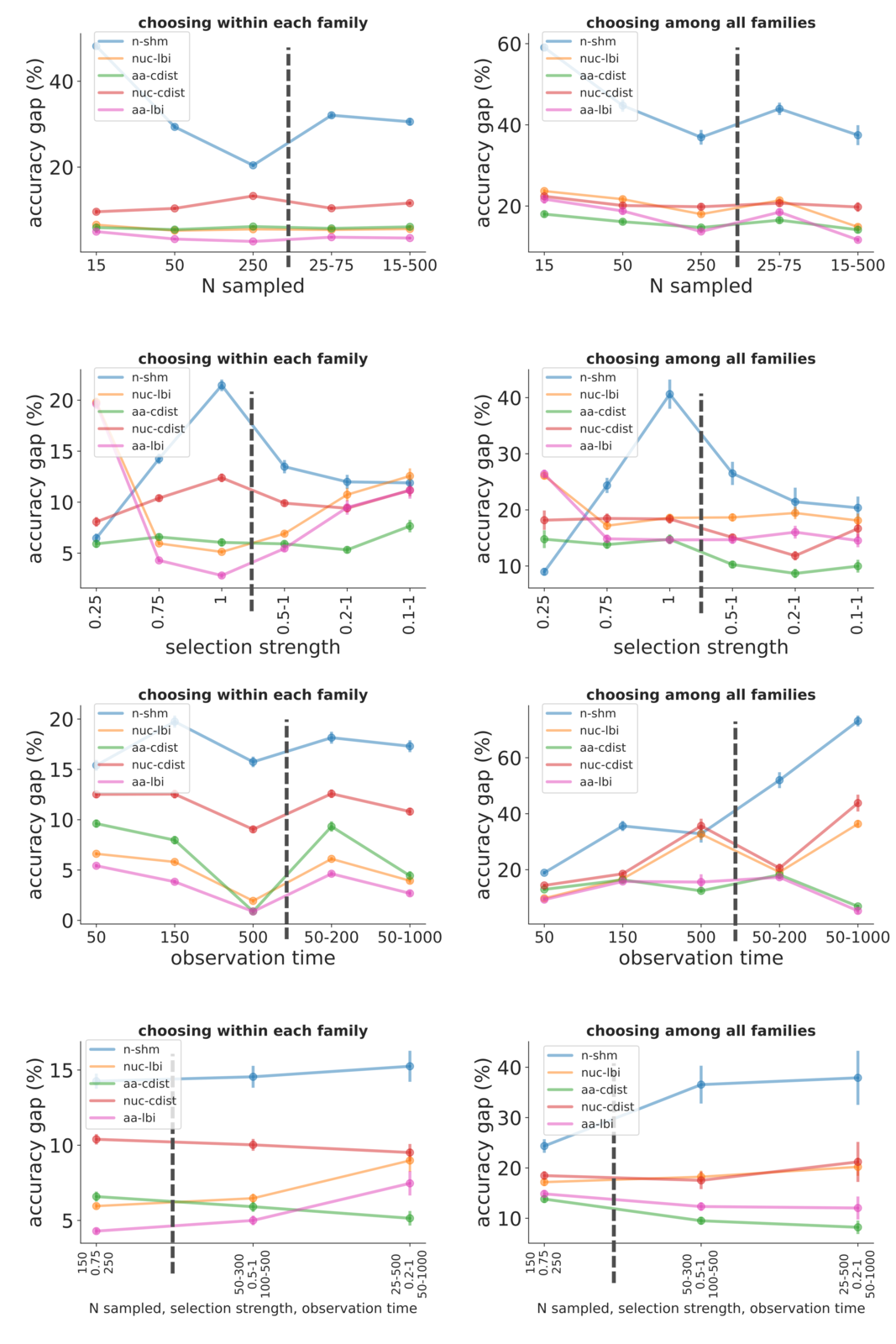

Supplement: S5 Fig — Within each plot, families in samples used to calculate points with x values to the left of the dashed line all have the same parameters, whereas those to the right have values sampled from the indicated range. For instance when varying N sampled sequences (top row), 15 sequences were sampled from every family in the leftmost points; whereas in making the rightmost points the number of sequences sampled from each family varied between 15 and 500. In the top three rows, we vary only one parameter at a time between families (N sampled, observation time, and selection strength), while in the bottom row we vary all three at once. (TIFF) [file pcbi.1008391.s005.tiff]

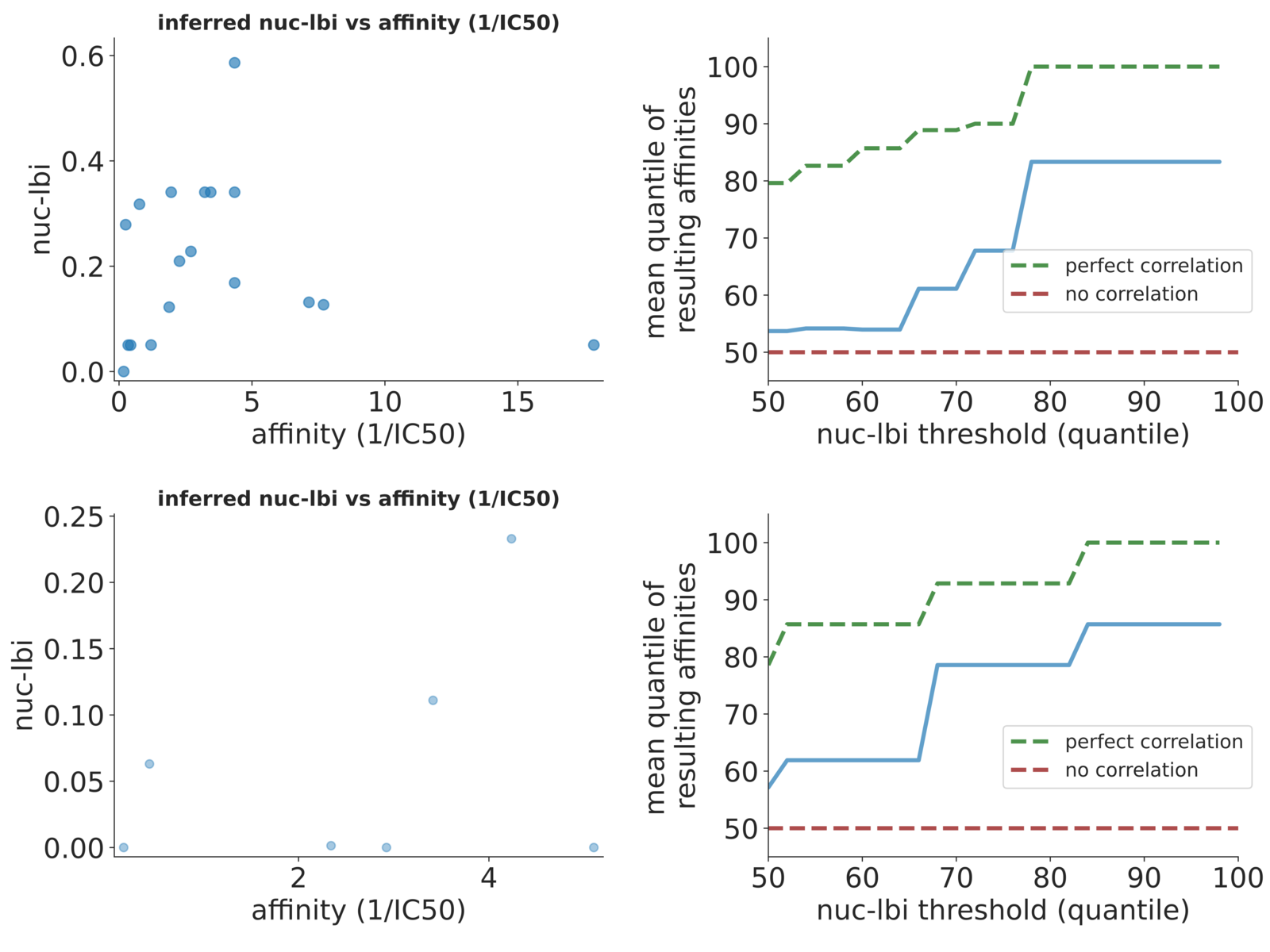

Supplement: S6 Fig — See caption to Fig 3. (TIFF) [file pcbi.1008391.s006.tiff]

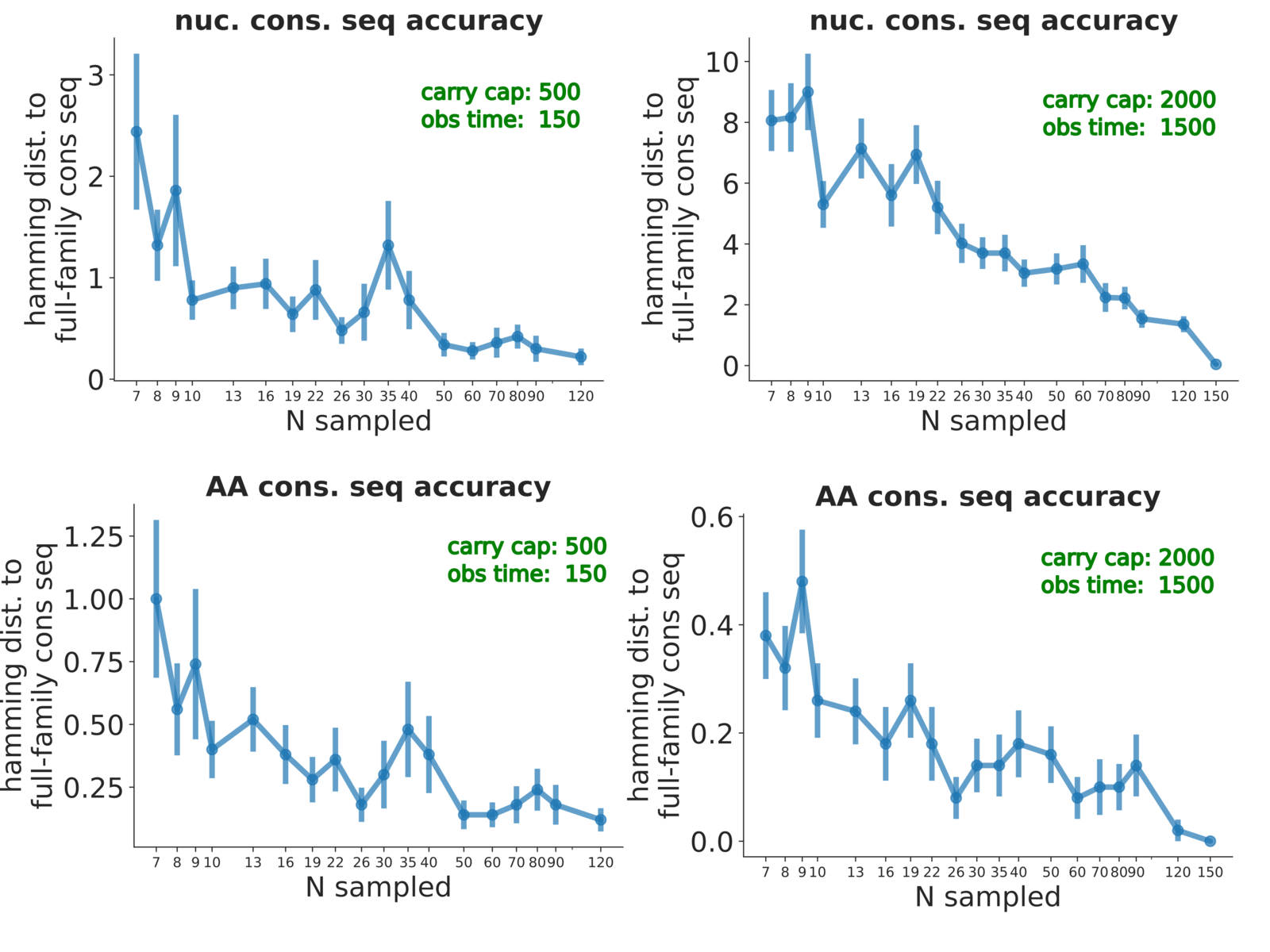

Supplement: S7 Fig — This shows the two extremes of parameters that seem to affect accuracy the most: very early times and small carrying capacities (left) vs very late times and large carrying capacities (right). Each point is the mean (± standard error) of 50 families, each with ≃150 sequences. The y value is the hamming distance (i.e. inaccuracy) between the consensus sequence calculated with the indicated number of sampled sequences (x axis) and the consensus sequence calculated on the entire family. (TIFF) [file pcbi.1008391.s007.tiff]

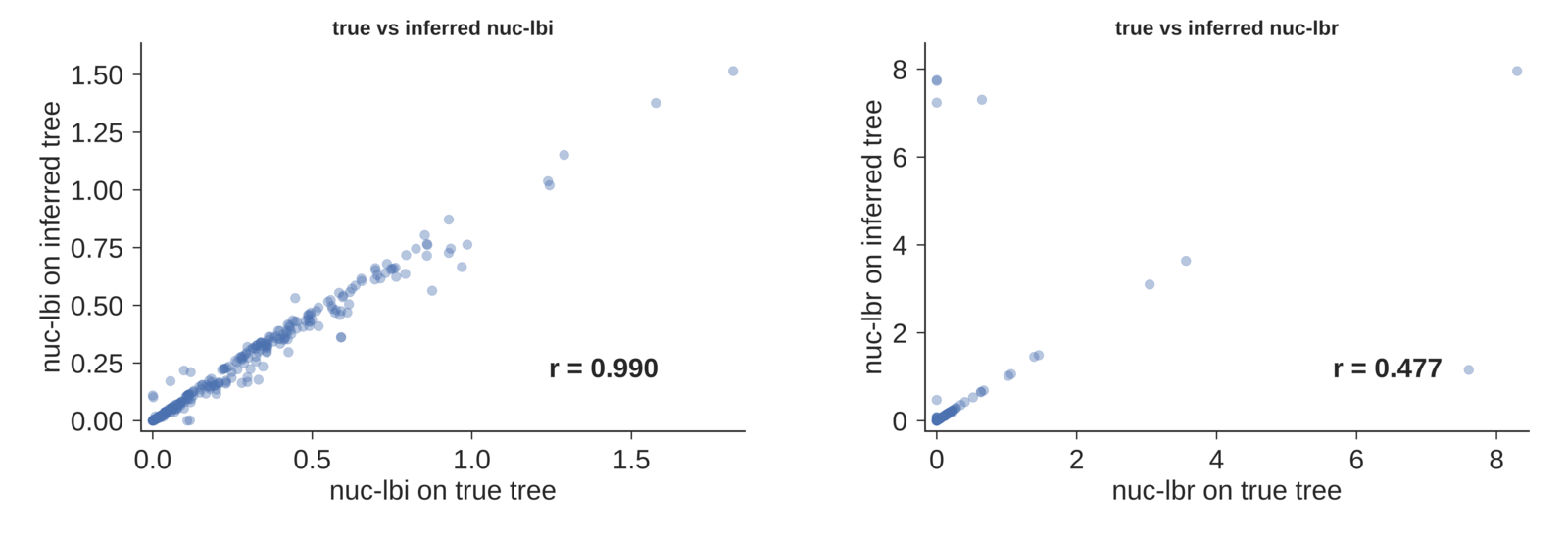

Supplement: S8 Fig — Inferred trees are made with the approximate, but very fast, method run by default partis (see Methods). The lefthand plot suggests that nuc-lbi is largely insensitive even to very heuristic tree inference. In the righthand plot, on the other hand, the handful of points with highly discrepant true and inferred values indicate that for nuc-lbr it is worth using a more sophisticated phylogenetic inference program if at all possible. (TIFF) [file pcbi.1008391.s008.tiff]

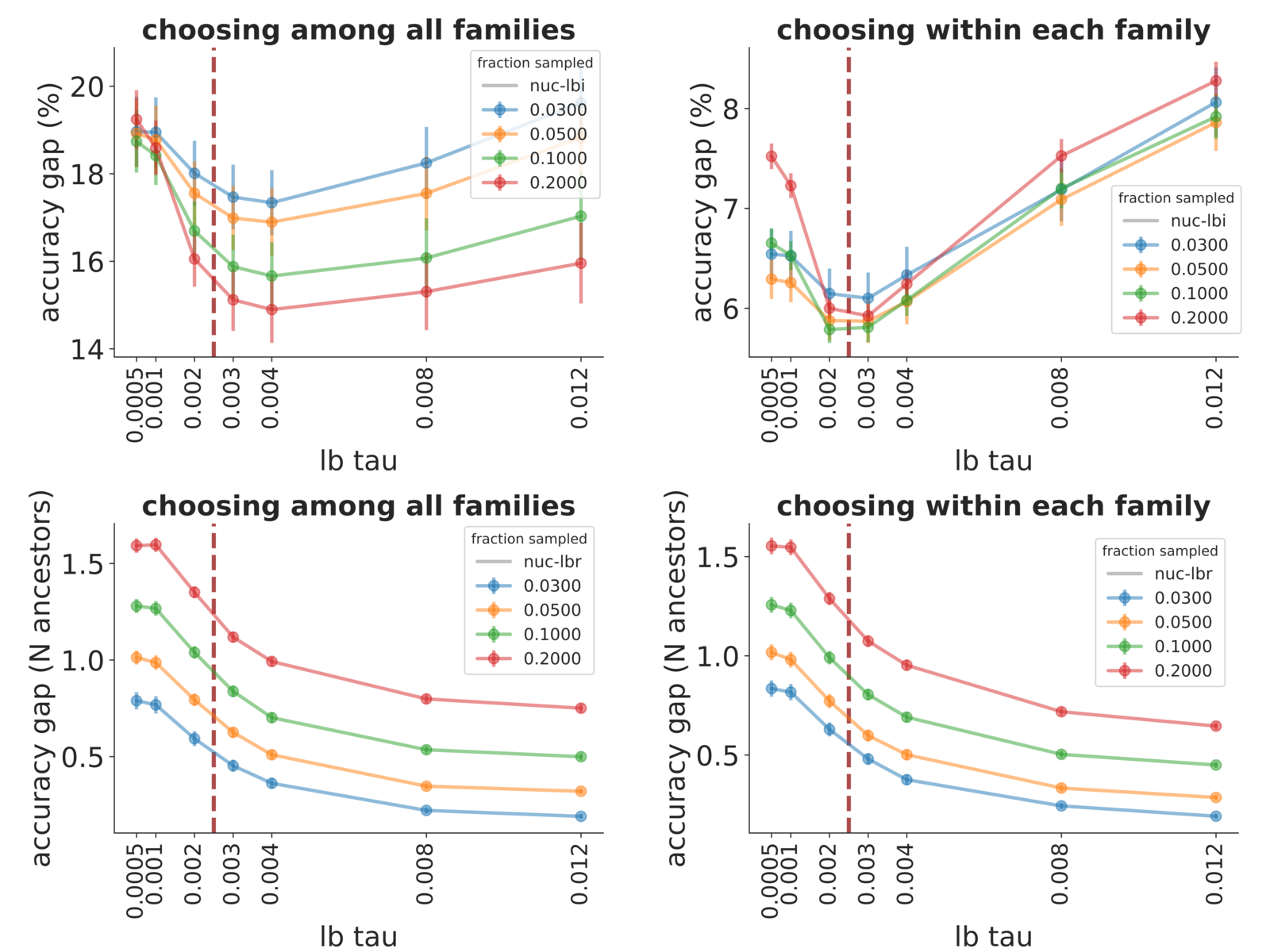

Supplement: S9 Fig — Dashed red line corresponds to the value expected from dimensional analysis 1/ℓseq = 1/400. The fraction observed corresponds to sampling between 30 and 200 sequences from a carrying capacity of 1000. Note that the vertical ordering of lines (i.e. whether performance is better for higher or lower sampling fractions) is not really informative in this plot—the order reverses depending on whether we sample ancestors or not, i.e. to a large extent it just measures the fraction of sampled sequences that are leaves. (TIFF) [file pcbi.1008391.s009.tiff]

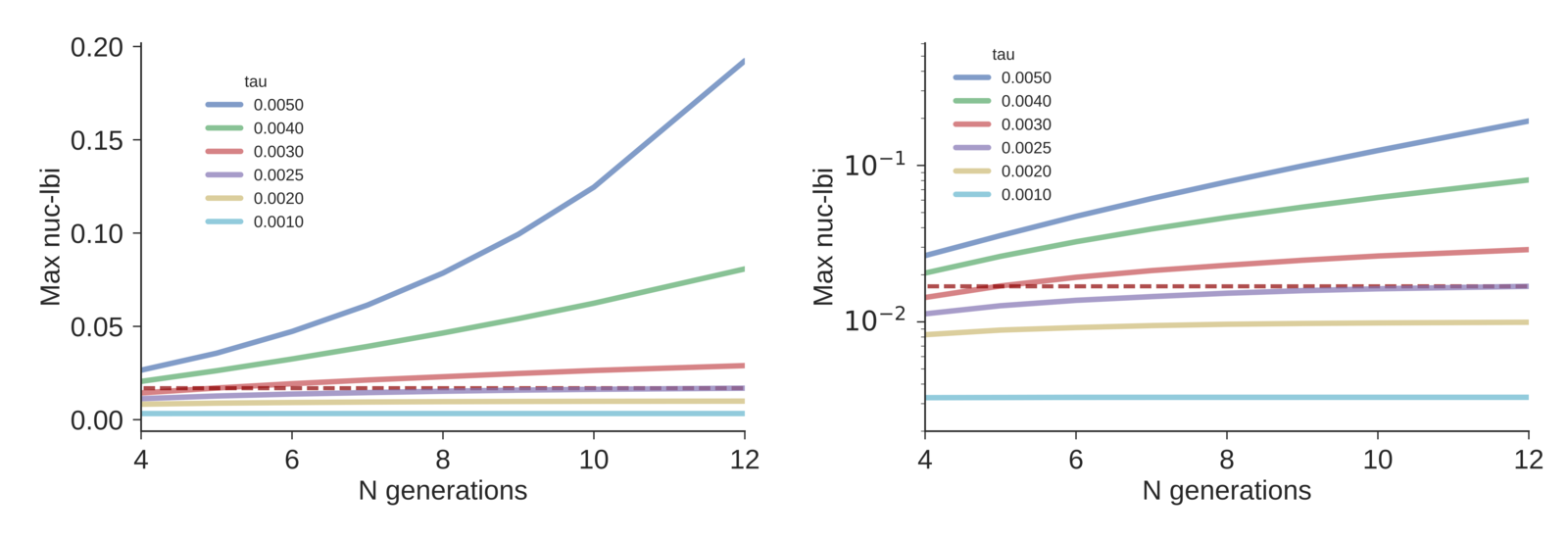

Supplement: S10 Fig — Plots show the maximum nuc-lbi value among the nodes in a particular reference synthetic “super branchy” tree as a function of tree depth (N generations). The asymptotic value for 1/ℓseq = 1/400 is shown in dashed red; the maximum nuc-lbi value converges to an asymptote for τ less than this, while for τ greater than this the maximum diverges. (TIFF) [file pcbi.1008391.s010.tiff]
